# Crosstalk of moderate ROS and PARP‐1 contributes to sustainable proliferation of conditionally reprogrammed keratinocytes

**DOI:** 10.1002/jbt.23262

**Published:** 2022-11-24

**Authors:** Linhua Liu, Abdul M. Mondal, Xuefeng Liu

**Affiliations:** ^1^ Center for Cell Reprogramming, Department of Pathology, Lombardi Comprehensive Cancer Center Georgetown University Medical Center Georgetown Washington USA; ^2^ Department of Environmental and Occupational Health Guangdong Medical University Guangdong Dongguan China; ^3^ Wexner Medical Center, Department of Pathology Ohio State University Columbus Ohio USA

**Keywords:** cell cycle, cell proliferation, conditionally reprogrammed cells (CRCs), human foreskin keratinocytes (HFK), hydroquinone, PARP‐1

## Abstract

Conditionally reprogrammed cell (CRC) technique is a promising model for biomedical and toxicological research. In the present study, our data first demonstrated an increased level of PARP‐1 in conditionally reprogrammed human foreskin keratinocytes (CR‐HFKs). We then found that PARP inhibitor ABT‐888 (ABT), reactive oxygen species (ROS) scavenger N‐acetyl‐l‐cysteine (NAC), or combination (ABT + NAC) were able to inhibit cell proliferation, ROS, PARP‐1, and ROS related protein, NRF2, and NOX1. Interestingly, knockdown of endogenous PARP‐1 significantly inhibited cell proliferation, indicating that the increased PARP‐1 expression was critical for CR. Importantly, we found that a moderate level of ROS contributed the cell proliferation and increased PARP‐1 since knockdown of PARP‐1 also inhibited the ROS. The similar inhibition of cell proliferation, ROS, and expression of PARP‐1 and NRF2 proteins was observed when CR‐HFKs were treated with hydroquinone (HQ), a key component from skin‐lightening products. Moreover, the treatment of HQ plus treatment of ABT, NAC, or combination can further inhibit cell proliferation, ROS, expression of PARP‐1, and NRF2 proteins. PARP‐1 knockdown inhibited the population doubling (PDL) and treatment of HQ inhibited the PDL further, as well as the change of ROS. Finally, we discovered that pathways including cyclin D1, NRF2, Rb and pRb, CHK2, and p53, were involved in cell proliferation inhibition with HQ. Taken together, our findings demonstrated that crosstalk between ROS and PARP‐1 involves in the cell proliferation in CR‐HFKs, and that inhibition of CR‐HFK proliferation with HQ is through modulating G1 cell cycle arrest.

## 1 INTRODUCTION

Cellular electrophiles, in general, are considered as detrimental to cells.^[^ ^1^ ^]^ However, both proliferating noncancerous and cancerous cells utilize these electrophiles for pro‐survival and cell cycle promoting signaling.^[^ ^2^, ^3^ ^]^ Reactive oxygen species (ROS) is the consequence of free radicals, including electrophiles.^[^ ^4^ ^]^ In a biological context, ROS are generated as a natural by‐product of the cellular metabolism of oxygen and play important roles in cell signaling and homeostasis.^[^ ^5^, ^6^ ^]^ A recent study showed that NOX1, a ROS‐generating oxidase, promotes the self‐renewal property of CD133+ thyroid cancer cells through activation of the Akt signaling.^[^ ^7^ ^]^ ROS are also produced in immune cell signaling via the NOX1 pathway.^[^ ^8^ ^]^ Phagocytic cells such as neutrophils, eosinophils, and mononuclear phagocytes produce ROS when they are stimulated.^[^ ^9^ ^]^ Therefore, the redox shift to mild oxidant release must be balanced by multiple defense mechanisms.^[^ ^2^ ^]^

Poly (ADP‐ribose) polymerase 1 (PARP‐1), an enzyme that catalyzes PARylation, is a key effector involved in homeostasis via DNA repair, replication, transcription, and genomic methylation, and is crucial for cell cycle control and cell proliferation.^[^ ^10^, ^11^, ^12^, ^13^ ^]^ PARP‐1 can be activated by a variety of stimuli or endogenous ROS.^[^ ^13^ ^]^ For example, hepatocytes from PARP‐1‐deficient mice show increased DNA damage and decreased proliferative responses to mitogens.^[^ ^14^ ^]^ PARP‐1 promotes the reprogramming process to generate high‐quality induced pluripotent stem cells (iPSCs) without c‐Myc.^[^ ^15^, ^16^ ^]^ These reports suggest that PARP‐1 and ROS may function as beneficial molecules or networks in the process of cell proliferation and reprogramming.

Recently we developed a cell technology named as conditional reprogramming (CR), coculturing target cells with irradiated feeder 3T3‐J2 fibroblasts (iJ2) in presence of a Rho kinase inhibitor. This technique enables normal and tumor primary epithelial cells to propagate indefinitely in vitro without genotype or phenotype alteration of the original cells. The resulting cells refer as conditional reprogramming cells (CRCs), which provide a promising cell model for biomedical and toxicological research.^[^ ^17^ ^]^ Since irradiation is a well‐known factor that can induce elevated ROS and activate PARP‐1 in vitro and in vivo,^[^ ^18^, ^19^, ^20^ ^]^ we hypothesize that crosstalk between ROS and PARP‐1 contributes to cell proliferation of conditionally reprogrammed human foreskin keratinocytes (CR‐HFKs).

In the present study, we demonstrated that crosstalk between moderate ROS and PARP‐1 regulates sustainable proliferation of CR‐HFK and inhibition with an antioxidant hydroquinone (HQ), a key component of skin‐lightening products.^[^ ^21^, ^22^ ^]^ We first observed an increased level of PARP‐1 in CR‐HFKs. Treatments with PARP inhibitor ABT‐888 (ABT), ROS scavenger N‐acetyl‐l‐cysteine (NAC), or combination (ABT + NAC) were able to inhibit cell proliferation, ROS, PARP‐1, and ROS related proteins, NRF2, and NOX1. Knockdown of endogenous PARP‐1 significantly inhibited cell proliferation. We also observed the similar inhibition of cell proliferation, ROS, and expression of PARP‐1 and NRF2 proteins when CR‐HFKs were treated with HQ. PARP‐1 knockdown inhibited the population doubling (PDL) and HQ inhibited the PDL further. Finally, we discovered that pathways including cyclin D1, NRF2, Rb and pRb, CHK2, and p53, were involved in cell proliferation inhibition with HQ. Taken together, our findings demonstrated that crosstalk between ROS and PARP‐1 involves in the cell proliferation in CR‐HFKs, and that inhibition of CR‐HFKs proliferation with HQ is through modulating G1 cell cycle arrest.

## 2 MATERIALS AND METHODS

### 2.1 Cell culture

The tissue specimens from neonatal foreskins were collected from the patients in accordance with the Institutional Review Board (IRB) protocols of Georgetown University (Protocol no: IRB 2002‐021).^[^ ^23^, ^24^ ^]^ Written informed consent from all the participants was taken before any clinical information or specimens were collected. Primary epithelial cells were isolated from the minced and digested tissues as described previously.^[^ ^17^ ^]^ The isolated primary HFKs were grown in CR culture system: F medium [3:1 (v/v) DMEM (Dulbecco's Modified Eagle Medium) and F‐12 nutrient mix] [containing 10% (v/v) fetal bovine serum] containing 0.125 ng/ml epidermal growth factor, 25 ng/ml hydrocortisone, 5 μg/ml insulin, 0.1 nM cholera toxin (Sigma‐Aldrich), 10 μg/ml gentamicin, 250 ng/ml amphotericin B (Gibco), and 10 μM Rho‐associated kinase (ROCK) inhibitor Y‐27632 (Enzo Life Sciences) in the presence of iJ2.^[^ ^23^ ^]^ CRCs were passaged at 1:8 when reached 80%–90% confluent. The viability of the cells was measured using trypan blue staining before every passage. The epithelial cells were harvested from coculture with iJ2 using two‐step trypsinization.^[^ ^23^ ^]^ Cells were also cultured in conditioned medium (CM) containing 10 μM Y‐27632 and in two non‐CR conditions (F medium with irradiated J2 feeders or F medium with Y‐27632) when needed. CM was prepared from iJ2 culture as previously.^[^ ^23^ ^]^ Population doublings (PDL) were calculated as [lg(final number of cells) – lg(initial number of cells)]/lg2.^[^ ^25^, ^26^ ^]^ For quantification of short‐term proliferation (within a week), culture cells were monitored in IncuCyte live‐cell analysis system using IncuCyte ZOOM software (Essen BioScience).

### 2.2 Chemical treatment

HFK cells, cultured in completed CM, were treated with HQ (at 2.5, 5, 10, 20, 40, and 80 μM), for 48 h. HFK cells treated with PBS were used as controls. Similarly, PARP inhibitor ABT and a ROS scavenger NAC were applied in this study at a concentration of 2.0 μM, and 500 μM, respectively.

### 2.3 Quantitative reverse transcription polymerase chain reaction

Total cellular RNA was isolated using the RNeasy Plus Mini Kit (Qiagen) according to the manufacturer's instructions. cDNAs were synthesized using SuperScript III First‐Strand Synthesis System Kit (Invitrogen), followed by Quantitative reverse transcription polymerase chain reaction (qRT‐PCR) using SsoAdvanced Universal SYBR Green Supermix (Bio‐Rad). Primers used for PARP‐1 were forward: 5′‐CCCCACGACTTTGGGATGAA‐3′ and reverse: 5′‐AGACTGTAGGCCACCTCGAT‐3′. The internal loading control was β2‐microglobulin (B2M) (forward 5′‐GGACTGGTCTTTCTATCTCTTGT‐3′; reverse 5′‐ACCTCCATGATGCTGCTTAC‐3′).

### 2.4 Immunoblotting

The harvested epithelial cells were lysed for total protein as previously described.^[^ ^26^, ^27^ ^]^ The primary antibodies used were: PARP‐1 (Santa Cruz Biotechnology, 1:200), NRF2 (Santa Cruz Biotechnology, 1:300), Cyclin D1 (Santa Cruz Biotechnology, 1:300), Cyclin D2 (Santa Cruz Biotechnology, 1:500), Cyclin B1 (Santa Cruz Biotechnology, 1:300), CDK4 (Santa Cruz Biotechnology, 1:500), p53/DO‐1 (Santa Cruz Biotechnology, 1:500), p21 (Santa Cruz Biotechnology, 1:500), p16 (Santa Cruz Biotechnology, 1:500), NOX1 (Abcam, 1:500), pRb (Cell Signaling Technology, 1:1000), Rb (Cell Signaling Technology, 1:2000), CHK2 (Cell Signaling Technology, 1:1000), Caspase‐3 (Cell Signaling Technology, 1:1000), Caspase‐7 (Cell Signaling Technology, 1:1000), and GAPDH (Santa Cruz Biotechnology, 1:10,000). The secondary antibodies (HRP‐conjugated anti‐mouse IgG or anti‐rabbit IgG) were purchased from Santa Cruz Biotechnology and used at 1:5000 dilution. The blots were quantified by ImageJ (https://imagej.en.softonic.com/).

### 2.5 PARP‐1 knockdown

PARP‐1 shRNA lentiviral particles (Santa Cruz Biotechnology) were used to knockdown endogenous expression of PARP‐1. Negative control lentiviral particles (Santa Cruz Biotechnology) were used as control. The transduction was performed according to the manufacturer's instructions. The viral transduced cells were selected by treating with 4 μg/ml puromycin. The control cells were referred to as shNC, and the PARP‐1 knockdown cells were referred to as shPARP‐1 in the experiment.

### 2.6 Intracellular ROS measurement

ROS levels were determined using the Reactive Oxygen Species Assay Kit (Abcam #ab113851) as per the manufacturer's protocol. Briefly, the iJ2 cells were cleared by quick trypsinization and then the attached HFK cells were washed twice with ice‐cold PBS. The cells were incubated with 6‐carboxy‐2, 7‐dichlorodihydrofluorescein diacetate (DCFH‐DA) at a final concentration of 20 μM for 40 min at 37°C in a dark area. The ROS levels were measured by a fluorescence microscope (EVOS).

### 2.7 Statistical analysis

The means ± SD were used to describe the data. One‐way ANOVA or a double‐sided Student's *t*‐test was employed to test the differences among the samples, and the Least Significant Difference *t*‐test was used for Post Hoc Multiple Comparisons. For all statistical comparisons, a *p* value < 0.05 was considered significant.

## 3 RESULTS

### 3.1 Increased PARP‐1 expression is associated with sustained cell proliferation of CR‐HFKs

**Figure 1:**
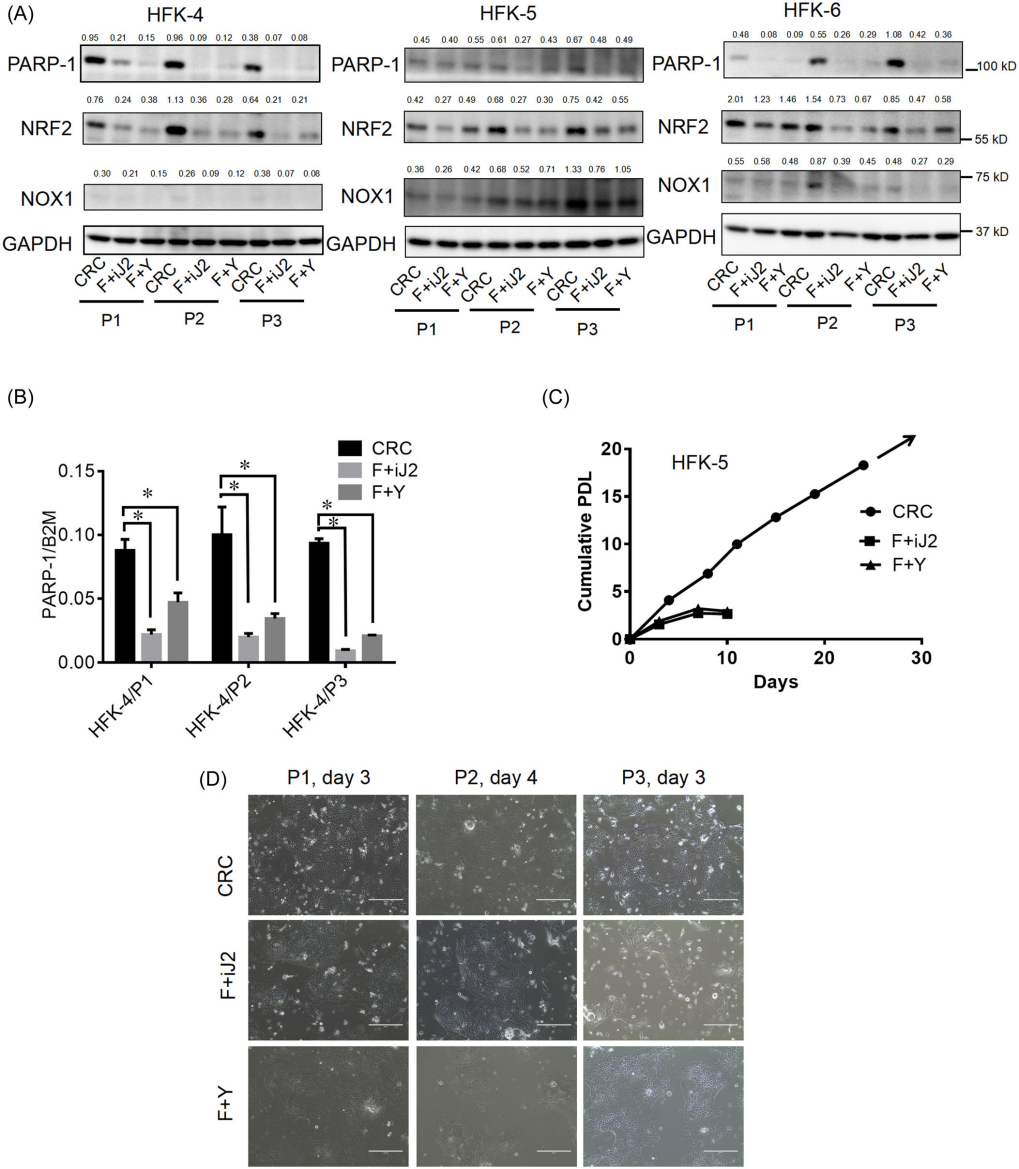
Sustained cell proliferation of CR‐HFKs correlates with increased PARP‐1 expression. (A) Protein expression for PARP‐1, NRF2, and NOX1 were measured by immunoblotting of three CR‐HFK lines (HFK‐4, HFK‐5, and HFK‐6 from three individual donors) cultured in conditional reprogramming condition and two non‐CR (F + iJ2 and F + Y) conditions at their P1, P2, and P3 passages. GAPDH was the loading control for quantification. (B) Expression of PARP‐1 mRNA in the cells was measured by qRT‐PCR. B2M mRNA was used for normalization. Data were mean ± SD from three independent experiments. (C) Representative growth curve for HFK‐5 cells cultured in CRC, F + iJ2, or F + Y until F + iJ2 and F + Y cells stopped growing. Cumulative PDLs were plotted versus time (days). (D) Representative images of the HFK‐5 at passages 1–3 in CRC, F + iJ2, or F + Y. Scale bars: 400 µm. Numbers above the blots were the quantitative expression for proteins. *, *p* < 0.05 versus the CRC in the passage.

Since PARP‐1 plays important roles in the regulation of ROS and promotes reprogramming process to generate the high quality of iPSCs,^[^ ^15^, ^16^ ^]^ we hypothesize that PARP‐1 is also critical in CR. We measured PARP‐1 expression in primary HFK cells grown in CR condition^[^ ^17^ ^]^ and compared with the cells grown in two non‐CR culture conditions: (i) F media with iJ2 cells, termed as F + iJ2, (ii) F media with ROCK inhibitor (Y‐27632), termed as F + Y (Figure 1A,B). As expected, non‐CR‐HFKs stopped proliferating after couple of passages while the CR‐HFKs continued to grow for long‐term in culture (Figure 1C,D). Interestingly, the CR‐HFKs showed increased levels of PARP‐1 expression both in protein (Figure 1A) and mRNA (Figure 1B) levels and remained high at different passages when compared to the non‐CR‐HFKs. These results suggested that increased PARP‐1 expression may contribute to the continued cell proliferation of HFKs under CR condition.

### 3.2 A moderate ROS level is essential for sustained cell proliferation of CR‐HFKs

**Figure 2:**
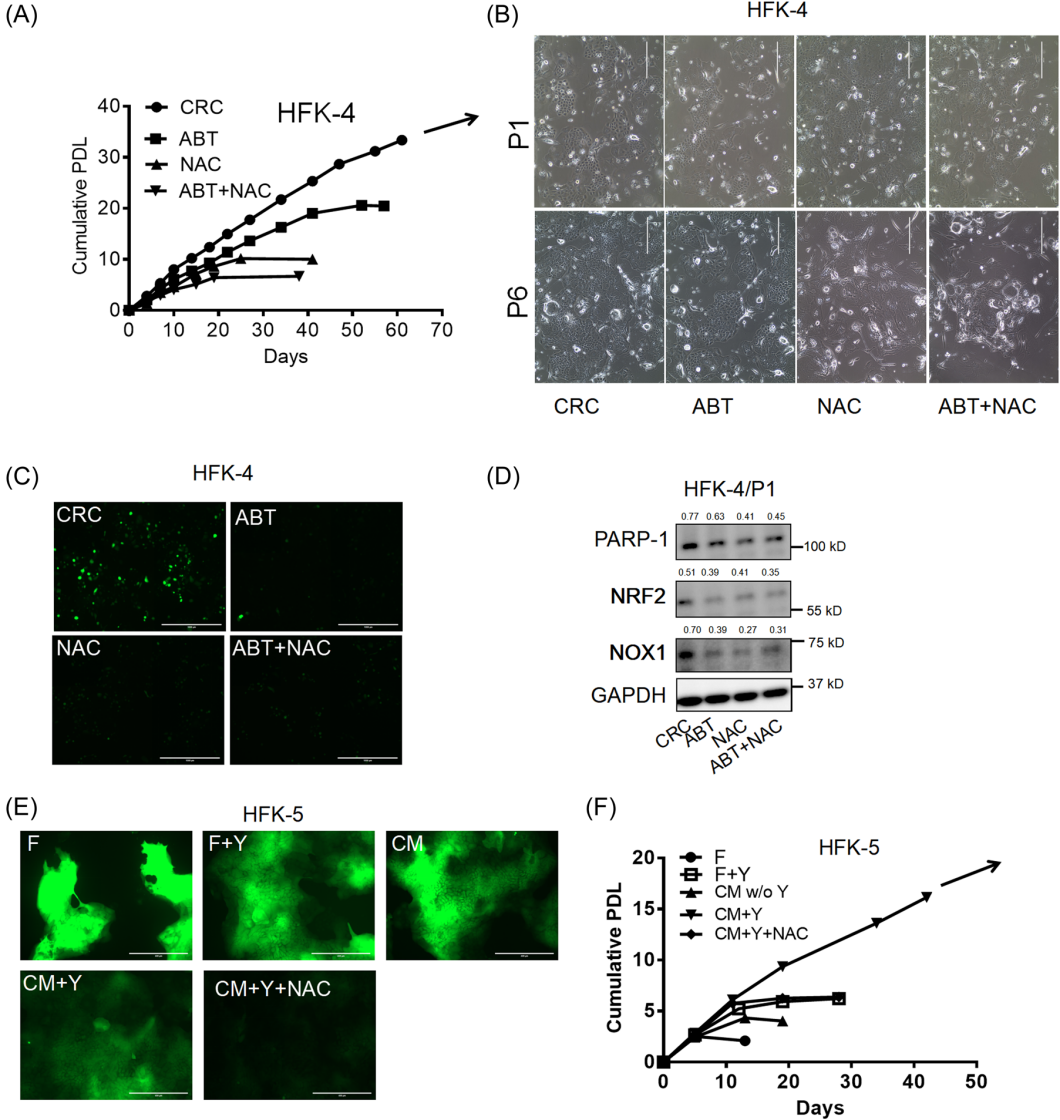
Moderate ROS levels support sustainable cell proliferation of CR‐HFKs. (A) Proliferation profile of the HFK‐4 cells in CRC in presence of ABT, NAC, or combination of both ABT and NAC. Cumulative PDLs were plotted versus time (days). (B) Representative images of the HFK‐4 at passage 1 (P1) and passage 6 (P6) in panel A. Scale bars: 400 µm. (C) Representative images to measure ROS levels in the HFK‐4 cells in CRC treated with ABT, NAC, or both ABT and NAC. Scale bars, 1000 µm. (D) Immunoblot analysis of PARP‐1 and two ROS‐related proteins, NOX1 and NRF2. GAPDH protein was the loading control. (E) ROS levels for HFK‐5 cells were measured in non‐CR medium (F, F + Y or CM) and CR medium (CM + Y) with or without NAC treatment. Scale bars: 200 µm. (F) Cumulative PDLs were calculated and plotted against days of the HFK‐5 cells cultured as shown in (E). Numbers above the blots were the quantitative expression for proteins.

To explore the role of PARP‐1 and ROS in contributing cell growth of the CR‐HFKs, we treated the cells with either a PARP inhibitor ABT or a ROS scavenger NAC or both. The optimum concentrations that showed inhibitory effect on cell proliferation without affecting cell viability were at 2 μM for ABT and 500 μM for NAC, respectively. CR‐HFKs treated with ABT or NAC exhibited proliferation inhibition and eventually ceased growth after 6–10 passages (Figure 2A,B). The inhibitory effect was more significant when the CR‐HFKss were treated with both ABT and NAC. Interestingly, ABT and NAC alone or in combination depleted the ROS levels of the CR‐HFKs (Figure 2C) and inhibited the expression of PARP‐1 protein, and two ROS‐related proteins, NOX1 (a ROS‐generating enzyme) and NFR2 (an antioxidant molecule; Figure 2D). These results suggested that ROS and PARP‐1 are associated with the indefinite proliferation of CR‐HFKs.

CM is the supernatant from iJ2 cells cultured in F media and can be used as a replacement of CR condition with iJ2 in coculture. Epithelial cells can be propagated indefinitely in complete‐CM (CM + Y), which has ample benefits for regenerative medicine and scientific research compared to cells cocultured with feeder cells.^[^ ^17^ ^]^ In this study, we also investigated ROS levels in HFKs in complete CM and other two non‐CR culture conditions. ROS levels were very high in the HFKs cultured in F medium (F), F medium with Y (F + Y) and in CM, whereas HFKs cultured in complete‐CM (CM + Y) showed a moderate level of ROS (Figure 2E). Most importantly, the HFKs cultured in CM + Y showed loss of ROS and eventually stopped dividing when NAC was used in the culture medium (CM + Y + NAC) (Figure 2E,F). Altogether, these results indicate that a moderate level of ROS, not at its highest levels, is critical for maintaining the indefinite growth of CR‐HFKs.

### 3.3 Endogenous PARP‐1 expression is critical to maintain moderate ROS levels and cell proliferation of CR‐HFKs

**Figure 3:**
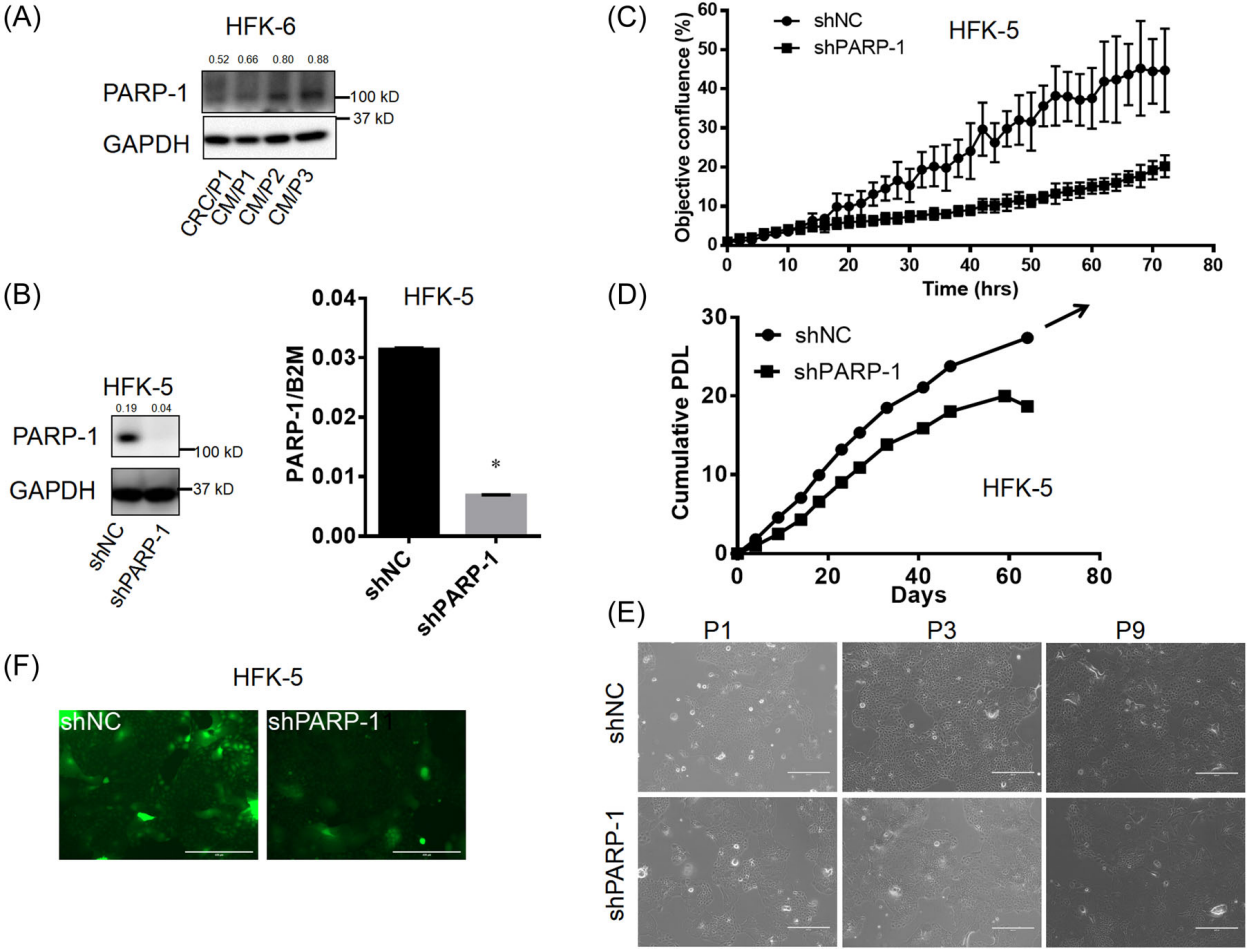
Knockdown of endogenous PARP‐1 inhibits cell proliferation and ROS levels. (A) PARP‐1 protein expression was compared by immunoblot analysis of HFK‐6 cells cultured in CRC or CM + Y. GAPDH was used as loading control. (B) Immunoblot analysis (left) and qRT‐PCR (right) for PARP‐1 expression in the shPARP‐1 lentiviral transduced HFK‐5 cells. GAPDH protein was used as loading control and B2M mRNA was used as internal control for normalization, respectively. (C) Proliferation of shPARP‐1 transduced cells was monitored for 3 days using IncuCyte. Data were mean ± S.D. from quadruplicate wells. (D) Long‐term proliferation profile of the puromycin‐selected shPARP‐1 cells compared to the negative control cells (shNC). Cumulative PDLs were calculated and plotted to days. (E) Representative images of the shPARP‐1 and shNC cells at passage 1 (P1), passage 3 (P3), and passage 9 (P9). Scale bars: 400 µm. (F) Representative images for ROS levels in the shNC and shPARP‐1 cells. ROS was measured by DCFDA staining. Scale bars: 200 µm. *, *p* < 0.05 versus the shPARP‐1. Numbers above the blots were the quantitative expression for proteins.

To investigate the role of PARP‐1 in CR‐HFKs, we first examined the PARP‐1 protein levels in CR‐HFKs under feeder‐free CR (CM + Y) and cocultured‐based CR conditions. Similar levels of PARP‐1 expression in both CR conditions were observed, indicating that PARP‐1 plays the similar role in maintaining cell proliferation of the CR‐HFKs (Figure 3A). Next, we depleted endogenous PARP‐1 expression by RNAi through shPARP‐1 lentiviral transduction (Figure 3B). The puromycin‐selected shPARP‐1 cells exhibited lower cell proliferation, while the negative control counterpart shNC cells continued to proliferate (Figure 3C). We next investigated the importance of PARP‐1 for the proliferation in long‐term culture. As expected, the shPARP‐1 cells stopped growing after 10 passages, while the shNC continued to proliferate indefinitely (Figure 3D,E). More interestingly, PARP‐1 depletion alleviated the ROS levels of the shPARP‐1 cells (Figure 3F). These results suggest that crosstalk between ROS and PARP‐1 involves in mainlining cell proliferation of the CR‐HFKs.

### 3.4 HQ‐mediated inhibition of ROS and PARP‐1 correlates with decreased cell proliferation

**Figure 4:**
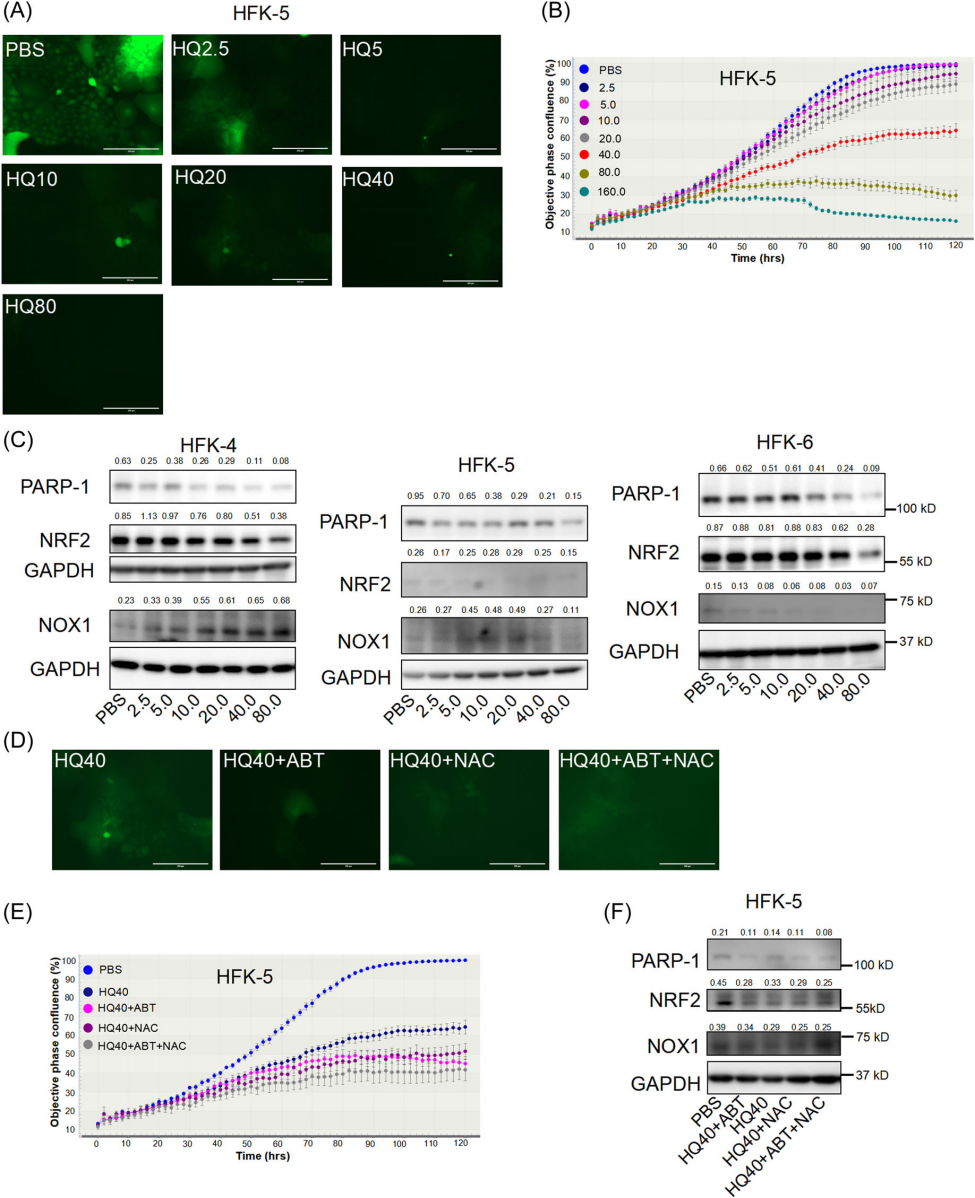
ROS and PARP‐1 inhibitions are associated with decreased cell proliferation of CR‐HFKs. (A) Representative images for ROS levels in the HFK‐5 CRCs treated with HQ at 2.5–80 μM final concentration. Only PBS was used as control. ROS was measured by DCFDA staining. (B) Cell proliferation was monitored for 5 days after HQ treatment using IncuCyte. (C) Immunoblot analysis for PARP‐1, NRF2, and NOX1 proteins in HFK‐4, HFK‐5, and HFK‐6 cells after treatment with HQ as mentioned in (A). GAPDH was used as loading control. (D) ROS levels were measured by DCFDA staining in the HFK‐5 cells treated with HQ with or without ABT and NAC as mentioned in the images. (E) Cell proliferation was monitored in IncuCyte for 5 days after HQ, ABT and NAC treatment as mentioned in panel E. Data were mean ± S.D. from quadruplicate wells. Scale bars: 200 µm. (F) Immunoblot analysis for PARP‐1, NRF2, and NOX1 in the HFK‐5 cells after HQ, ABT, and NAC treatment as mentioned in (E). GAPDH was used as loading control. Numbers above the blots were the quantitative expression for proteins.

HQ is an antioxidant and a skin‐bleaching agent. We chose HQ to investigate the role of ROS and PARP‐1 in cell proliferation of CR‐HFKs. Several studies revealed that HQ can inhibit cell growth via increasing ROS and DNA damage to cells.^[^ ^28^ ^]^ However, we observed that HQ inhibited ROS levels and caused proliferation inhibition of CR‐HFKs cultured in CM + Y in a dose‐dependent manner (Figure 4A,B). These results indicated that ROS plays a critical role in cell proliferation of primary HFK cells in CR condition. We next measured the expression of PARP‐1 and ROS‐related proteins, NRF2 and NOX1 in three isolates of CR‐HFKs treated with HQ. As expected, all three isolates of CR‐HFKs exhibited decreased levels of PARP‐1 and NRF2 expression with HQ treatment (Figure 4C). NOX1 expression did not show consistent dose‐dependent change in three CR‐HFKs (Figure 4C). However, expression of NRF2 and NOX1 remained at consistently high levels in the CR‐HFKs of all three isolates as compared to their non‐CR counterparts F + iJ2 and F + Y cells (Figure 1A). We also examined the effect of ABT and NAC in parallel to HQ treatment in the CR‐HFKs. The moderate ROS levels (Figure 2E) decreased in treatment with HQ and loss of ROS was further aggravated with the treatment of ABT and NAC (Figure 4D). Interestingly, the CR‐HFKs showed decreased cell proliferation when treated with either HQ alone or in combination with ABT and/or NAC (Figure 4E). Most importantly, lack of cell proliferation of CR‐HFKs was associated with loss of expression of PARP‐1, NRF2 and NOX1 proteins (Figure 4F). Altogether, the data suggested that a crosstalk between ROS and PARP‐1 plays a critical role in sustained cell proliferation of cocultured‐based or CM‐based CR‐HFKs.

### 3.5 Knockdown of PARP‐1 mediates inhibition of cell proliferation through depletion of ROS

**Figure 5:**
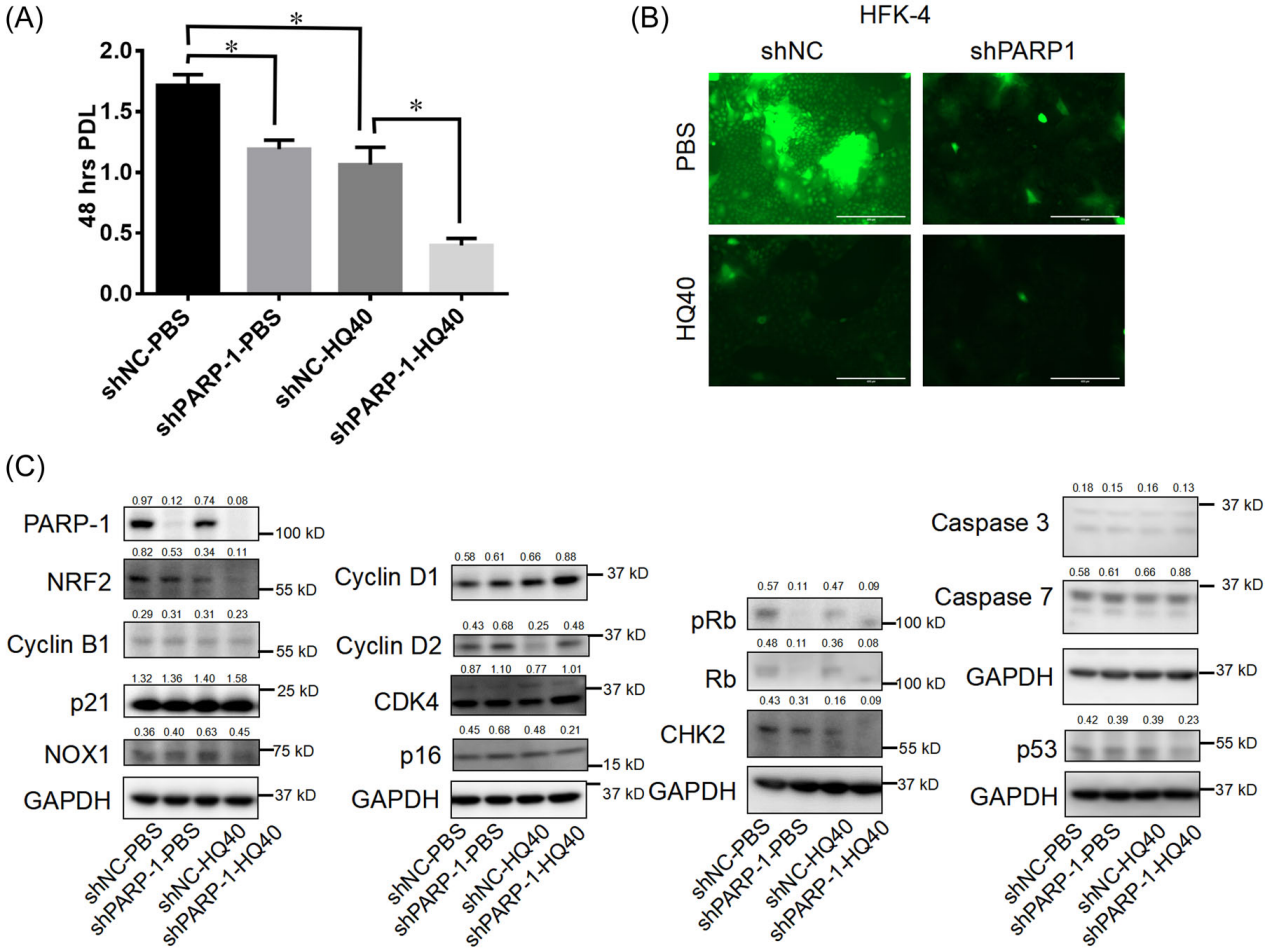
Knockdown of PARP‐1 mediates cell proliferation inhibition via depletion of ROS. (A) PDLs were measured in the shNC and shPARP‐1 CRCs after HQ treatment (at 40 μM) for 48 h. PBS only was used as control for both cells. (B) Representative images for ROS levels of the shNC and shPARP‐1 cells as mentioned in (A). Scale bars: 200 µm. (C) Immunoblot analysis for PARP‐1, NRF2, Cyclin B1, 21, NOX1 Cyclin D1, Cyclin D2, CDK4, p16, Rb, pRb, CHK2, Caspase 3, Caspase 7, and p53 in the shNC and shPARP‐1 cells treated with or without HQ. GAPDH was the loading control. Numbers above the blots were the quantitative expression for proteins. *, *p* < 0.05.

To delineate the molecular pathways in regulating cell proliferation of CR‐HFKs and effects of knockdown of PARP‐1 and/or HQ treatment, we examined the shRNA lentiviral transduced CR‐HFK‐4 isolate in CM + Y. As shown in Figure 3C,D, the shPARP‐1 cells exhibited a decreased PDL compared to the control shNC transduced cells (Figure 5A). The proliferation inhibition was more significant when the shPARP‐1 cells were treated with HQ (Figure 5A). Interestingly, ROS levels in the shPARP‐1 cells were also further inhibited in HQ‐treated cells (Figure 5B). These results supported that ROS plays a critical role in cell proliferation of CR‐HFKs.

To explore the underlying molecular mechanism of ROS‐driven cell proliferation, we examined the shPARP‐1 cells treated with or without HQ for expression levels of various molecules: cell cycle checkpoint (cyclin B1, cyclin D1, cyclin D2, CDK4, and CHK2), p53/Rb pathway (Rb, pRb, p53, p21, and p16) and death‐receptor apoptotic pathway (caspase‐3 and caspase‐7) as well as antioxidant related molecules NRF2 and NOX1 (Figure 5C). As expected, NRF2 expression was inhibited in the shPARP‐1 and was further inhibited when the cells were treated with HQ compared to the shNC control cells. Interestingly, consistent changes of shared pathways including cyclin D1, CHK2, p53, Rb, and pRb were observed both in the HQ‐treated shNC and shPARP‐1 cells. Thus, our results suggested that ROS and anti‐oxidation machinery are involved in the cell proliferation retardation mediated by PARP‐1 knockdown in CR‐HFKs or in the cells treated with HQ, the underlying mechanism is by modulating the G1 cell cycle arrest.

## 4 DISCUSSION

In this study, we found that the crosstalk between PARP‐1 and moderate ROS levels is involved in cell proliferation of CRCs with or without treatment of HQ. Irradiation can intrigue oxidative stress and DNA damage to cells by elevating the ROS level, thus irradiation is a detrimental factor to organisms.^[^ ^29^ ^]^ In general, cancer cells take advantage of ROS to maintain self‐renewal activity and participate in oncogenesis processes. We established CR‐HFKs to further explore the role of crosstalk between PARP‐1 and ROS. CR‐HFKs exhibited an increased PARP‐1 level and indefinite cell proliferation, while treatment of ABT or NAC or both inhibited cell proliferation concomitant with ROS and downregulated expression of PARP‐1, NRF2, NOX1. Interestingly, we also found that the ROS level did not correlate positively with the cell proliferation and PARP‐1 expression, only moderate ROS levels contributed to indefinite cell proliferation. Our data demonstrated that mild ROS can be turned into beneficial factor to CR‐HFKs, and PARP‐1 mediated antioxidative machinery plays pivotal role during this process.

Redox biological reactions are now accepted to bear the Janus faceted feature of promoting both physiological signaling responses and pathophysiological cues. Endogenous antioxidant molecules participate in both scenarios.^[^ ^30^ ^]^ Due to pressure induced by an elevated and sustainable redox shift to a mild oxidative environment, cells have developed efficient mechanisms of adaptation and functional transformation of “bad” to “good” molecules, which promote cell proliferation and survival at different signaling levels.^[^ ^30^ ^]^ PARP‐1 is a multifunctional enzyme that regulates many intracellular processes, including DNA repair, metabolism, signaling, and transcription, by direct interactions with other proteins and DNA, these interactions are related to their ADP‐ribosylation and auto‐ADP‐ribosylation of PARP‐1.^[^ ^2^, ^31^, ^32^ ^]^ Cell cycle progression depends on PARP‐1 transcription.^[^ ^31^ ^]^ A recent study revealed that cell arrest in G1 or exit to G0 leads to PARP‐1 repression by Rb‐based multiprotein complexes, which are also known to repress transcription of E2F‐dependent genes responsible for cell transition to S phase.^[^ ^2^ ^]^ In our study, we found that PARP‐1 knockdown inhibited cell growth in the short term and abrogated indefinite cell proliferation in the long‐term cultures. Treatment of HQ inhibited PARP‐1 expression and ROS level, followed by cell growth retardation. Inhibition of PARP‐1, ROS, and cell growth was observed with treatment of ABT, NAC, or combination. Importantly, we found that ROS is a pathway shared in CR‐HFKs with and without treatment of HQ, since PARP‐1 knockdown inhibited ROS level and further inhibition was obtained in CR‐HFKs treated with HQ. A study also found that ROS is inhibited by PARP‐1 knockout and plays a key role in cell apoptosis induced by irradiation in mice.^[^ ^33^ ^]^

It has been reported that PARP‐1 contributes to early‐stage epigenetic modification during somatic cell reprogramming and can promote reprogramming and maintain pluripotency.^[^ ^34^ ^]^ Oct‐4/Sox2/Klf4/PARP‐1 (OSKP)‐derived iPSC exhibited regular pluripotent properties, long‐term passages, and a more stable cellular‐divided period.^[^ ^15^ ^]^ PARP‐1 promoted the reprogramming process to generate a high quality of iPSCs, which may be used as a high‐quality and stable resource of hepatocytes.^[^ ^15^ ^]^ These implied a critical role of PARP‐1 in indefinite cell proliferation. PARP‐1 participates in a broad range of critical cellular processes including chromatin remodeling, DNA repair, genome integrity, and cell death. It executes its multi‐function by modulating gene expression.^[^ ^31^, ^32^, ^35^ ^]^ Exogenous stimulus, like mutagen, can induce ROS elevation and activation of PARP‐1. Nonetheless, oxidative insults may induce bulk macroautophagy with the accumulation of autophagosomes and autolysosomes with remarkable elevation of ROS, the overload of intracellular calcium, and robust depolarization of mitochondrial membrane potential, while mitochondria respiratory function is impaired and widespread mitophagy compromised cell viability.^[^ ^36^ ^]^ These are related to p53 transcriptional regulation,^[^ ^18^, ^37^ ^]^ however it does not seem that our study supports this, since we observed the antioxidative molecule, NRF2, and Rb/cyclin D1 mediated G1 cell cycle arrest.

In conclusion, we demonstrated that ROS and anti‐oxidation machinery are involved in cell proliferation retardation mediated by PARP‐1 knockdown in CR‐HFKs and cells treated with HQ. Our findings indicate that crosstalk between PARP‐1 and ROS plays a key role in cell proliferation, providing a new insight into CR research and its applications.

## AUTHOR CONTRIBUTIONS

*Conceptualization*: Linhua Liu. *Methodology*: Linhua Liu and Abdul M. Mondal. *Software*: Abdul M. Mondal. *Validation*: Xuefeng Liu. *Resources*: Xuefeng Liu. *Data curation*: Abdul M. Mondal. *Writing–original draft*: Linhua Liu. *Writing–review and editing*: Abdul M. Mondal and Xuefeng Liu. *Visualization*: Linhua Liu. *Supervision*: Xuefeng Liu. *Project administration*: Xuefeng Liu. *Funding acquisition*: Xuefeng Liu. All authors have read and agreed to the published version of the manuscript.

## CONFLICTS OF INTEREST

Several patents for conditional reprogramming technology have been awarded to Georgetown University by the United States Patent Office. The license for this technology has been given to a Maryland‐based start‐up company for commercialization. The inventor, X.L., and Georgetown University receive potential royalties and payments from the company. Other authors declare that they have no known competing financial interests or personal relationships that could have appeared to influence the work reported in this paper.

## Supporting information

Supporting information.

## Data Availability

The data that support the findings of this study are available from the corresponding author upon reasonable request.
