# Supplementary figures and images for: Crosstalk of moderate ROS and PARP‐1 contributes to sustainable proliferation of conditionally reprogrammed keratinocytes
Source: J Biochem Mol Toxicol. 2022 Nov 24;37(2):e23262. doi: 10.1002/jbt.23262 (PMC10078201; doi:10.1002/jbt.23262)

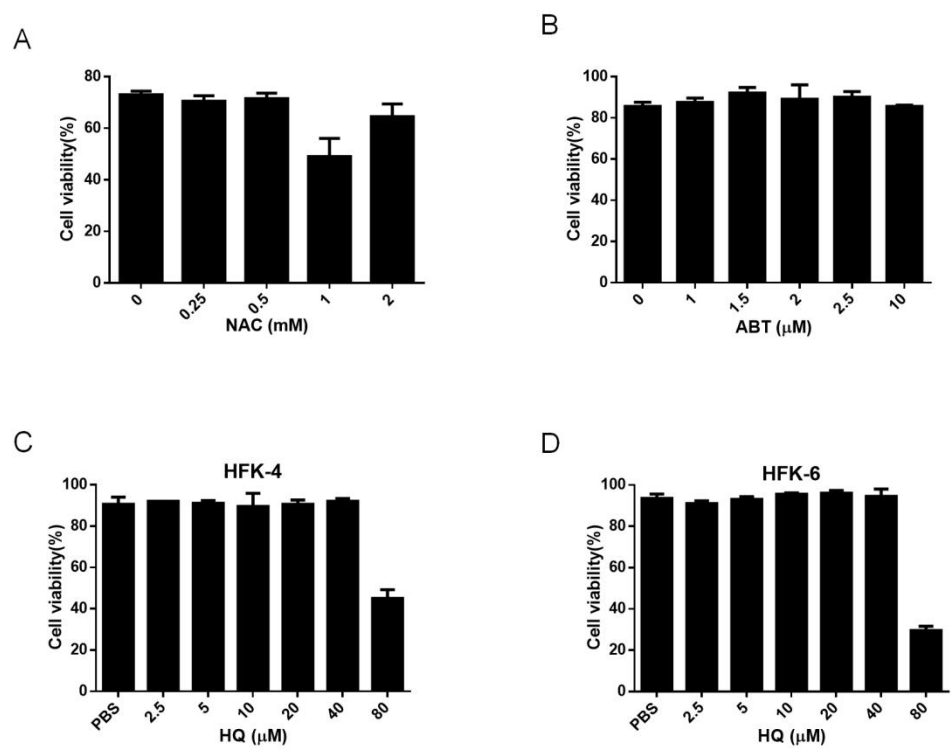

Fig. S1 Changes of cell viability of HFK cells induced by NAC, ABT, or HQ exposure for 72 hrs.

Supplement: Supplementary file 1 — Supporting information. [file JBT-37-0-s001.pdf]
